# A systematic approach to quantitative nanoscale mass spectrometry using atomic force microscopy

**DOI:** 10.1016/j.isci.2026.117900

**Published:** 2026-10-15

**Authors:** Sangwoo Kwon, Kyung Sook Kim

**Affiliations:** 1 Department of Biomedical Engineering, College of Medicine, Kyung Hee University, Seoul 02447, Republic of Korea; 2 Department of Biomedical Engineering, Graduate School, Kyung Hee University, Seoul 02447, Republic of Korea

**Keywords:** atomic force microscopy, microcantilever sensing, nanogram-scale mass detection, resonance frequency shift, finite-element simulation

## Abstract

Atomic force microscopy (AFM) enables mass sensing through resonance-frequency changes of microcantilevers, but quantitative measurements can be affected by calibration and environmental factors. Here, we investigated whether a calibrated AFM cantilever and controlled mass loading could provide nanogram-scale mass estimation. Cantilevers were cleaned and calibrated by force-distance measurements, and resonance acquisition parameters were optimized under ambient conditions. UV-curable resin droplets were locally deposited on the cantilever to generate mass loading, and resonance-frequency shifts were measured and analyzed using a mass-spring model and finite-element simulations. Increasing resin mass produced progressive decreases in resonance frequency, demonstrating a consistent relationship between mass loading and cantilever resonance behavior. Finite-element simulations reproduced the resonance responses and supported the mass estimates obtained from the experimental measurements. These findings establish a calibration and modeling framework for resonance-based mass quantification using conventional AFM cantilevers and provide a basis for extending quantitative mass analysis to microstructures and biological assemblies.

## Highlights

• AFM cantilever resonance frequency was systematically calibrated for mass sensing
• Localized resin loading produced measurable resonance shifts at nanogram masses
• Experimental resonance shifts agreed closely with COMSOL finite-element simulations
• The framework enables quantitative AFM mass analysis at the nanoscale

## Graphical abstract

**Figure:**
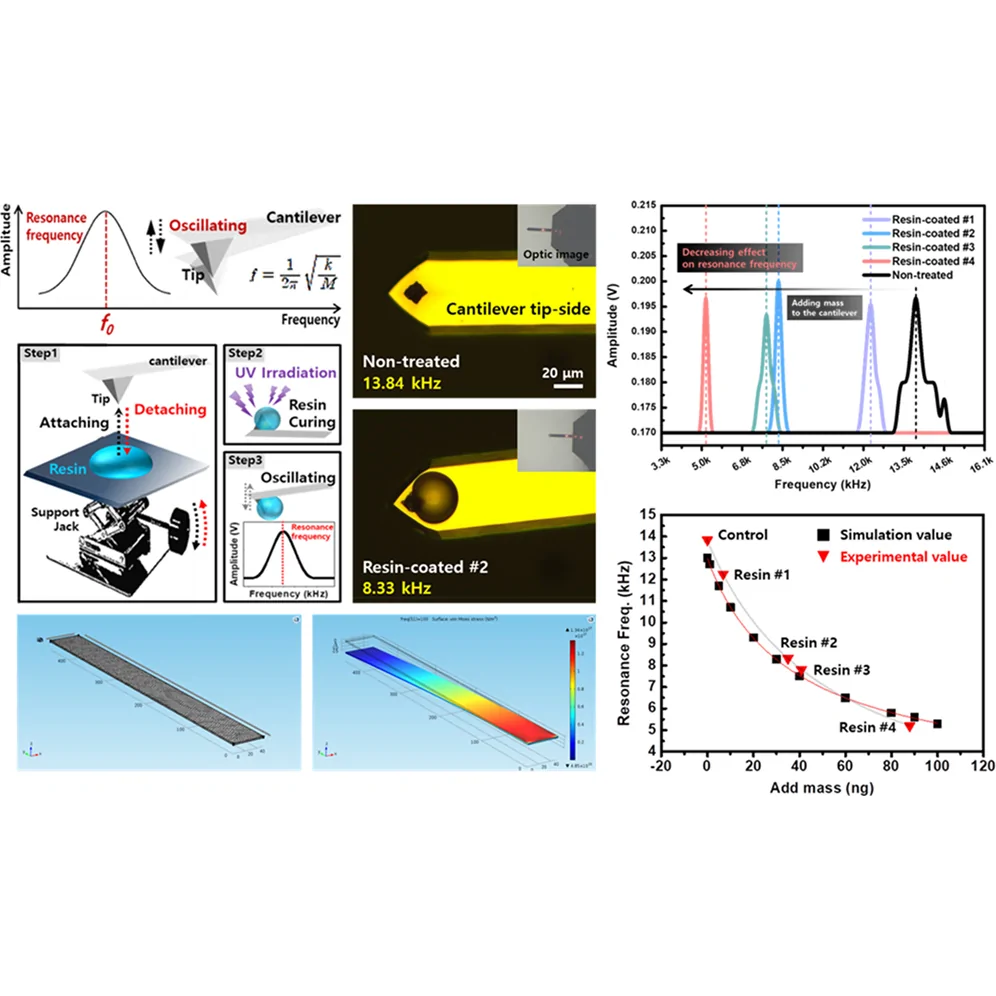

## Teaser

Sensor; Nanotechnology fabrication; Materials characterization

## Introduction

Atomic force microscopy (AFM) has traditionally been recognized as a powerful nanoscale imaging technique for characterizing surface topography. However, modern AFM platforms also provide a wide range of quantitative measurement capabilities, including force spectroscopy, nanomechanical mapping, stiffness characterization, electrical property analysis, and surface interaction measurements.^1^ Recent developments have further enabled quantitative evaluation of material properties such as Young’s modulus, adhesion force, viscoelasticity, and interfacial mechanics at the nanoscale. Consequently, AFM has evolved from a purely imaging-based technique into a versatile quantitative characterization platform for materials science, nanotechnology, and biological research.^2^

AFM has emerged as a powerful platform capable of detecting ultra-fine changes in mass, with sensitivity extending to nanogram (ng) and even sub-nanogram ranges. This capability is based on the fundamental operating principle of monitoring changes in the resonance frequency of a microcantilever when additional mass is introduced to its surface.^3^ Even minute quantities of adsorbed or attached substances alter the effective mass of the cantilever, inducing a measurable downward shift in resonance.^4^ This positions AFM as an exceptionally sensitive probe for nanoscale detection, enabling sensitivity levels inaccessible by conventional analytical methods.^5^^,^^6^

AFM’s nanoscale sensing capabilities have opened new fields of research in areas such as biosensing, materials science, and biomedical research. In biosensing, AFM cantilevers are used to quantify molecular binding events,^7^^,^^8^ protein aggregation,^9^ and nucleic acid hybridization without labels or invasive procedures.^10^^,^^11^ In materials science, nanogram detection allows for real-time monitoring of deposition processes,^12^ thin-film growth,^13^ and nanostructure assembly.^14^ In biomedical research, AFM enables the exploration of minute mass differences within cell aggregates, spheroids, and microtissues, proposing it as a quantitative platform for nanoscale dynamic mass analysis beyond mere imaging.^15^^,^^16^

Microcantilever-based resonance sensing has been extensively investigated for ultra-fine mass detection in biological, environmental, and materials-science applications. Recent studies have demonstrated enhanced sensitivity using two-dimensional material-coated cantilevers, monolithically integrated biosensor systems, and microelectromechanical systems (MEMS) piezoresistive cantilever platforms for nanoparticle and biomolecule detection. However, quantitative reproducibility remains challenging due to calibration uncertainty, environmental damping, and limited integration between experimental resonance analysis and computational validation. In this study, a systematic AFM-based calibration-to-validation workflow was established to improve the quantitative reliability of nanoscale resonance-based mass estimation using commercially available cantilever systems.^17^^,^^18^^,^^19^

However, several challenges impede nanogram-level detection. Reliance on nominal cantilever specifications or unverified calibration methods can lead to systematic errors. Damping in air, thermal drift, and laser alignment issues can obscure small frequency shifts.^20^^,^^21^ Moreover, achieving close alignment between experimental results and predictions of intrinsic frequency and mass loading effects is not straightforward.^22^^,^^23^^,^^24^^,^^25^^,^^26^ Addressing these issues necessitates a systematic methodology integrating careful cantilever preparation, rigorous calibration, controlled mass loading, and advanced computational modeling.^27^^,^^28^^,^^29^^,^^30^

This study addresses these challenges by establishing an integrated framework for nanogram-level mass detection using an AFM cantilever. Cantilevers were meticulously cleaned to eliminate extraneous contamination and directly calibrated using force-distance (FD) analysis.^1^ Accurate calibration of cantilever spring constants is essential for translating frequency shifts into effective resonance-based mass estimation values. Localized resin droplets were then deposited at the distal tip to introduce reproducible mass increments under ambient conditions, enabling precise control over effective loading. Resonance frequency shifts were quantified and systematically compared with high-fidelity finite-element simulations conducted in COMSOL Multiphysics® (COMSOL, Inc.), designed to capture localized perturbations at fine spatial resolution.

Through this combined approach, we derive a robust quantitative relationship between resonance frequency shifts and absolute nanogram mass variations. Close agreement between experimental, theoretical, and numerical simulations demonstrates both the accuracy and reproducibility of this methodology. By mitigating uncertainties related to calibration, environmental damping, and parameter optimization, this study expands the potential for utilizing AFM cantilevers as quantitative nanogram-level mass sensors. Beyond fundamental material characterization, this precision offers groundbreaking insights into cell mechanics, tissue composition, and disease progression by detecting minute mass differences within complex biological systems.

## Results

### Verification of AFM resonance frequency measurements in air conditions

Optimizing measurement conditions is crucial for obtaining precise resonance frequencies of AFM cantilevers. This requires a comprehensive calibration and measurement process. This process begins with dimensional calibration to accurately represent displacements in nanometers and micrometers. The dimensional calibration of the AFM’s horizontal and vertical axis scans was performed using calibration sample (Figure 1A). The calibration sample was patterned with dimensions of 10 × 10 × 0.085 μm^3^ in the *x*, *y*, and *z* axes, respectively. This enabled accurate representation of displacements in the nanometer and micrometer ranges in both two- and three-dimensional imaging modes. This step is crucial for minimizing geometric distortion during subsequent frequency analysis.

**Figure 1:**
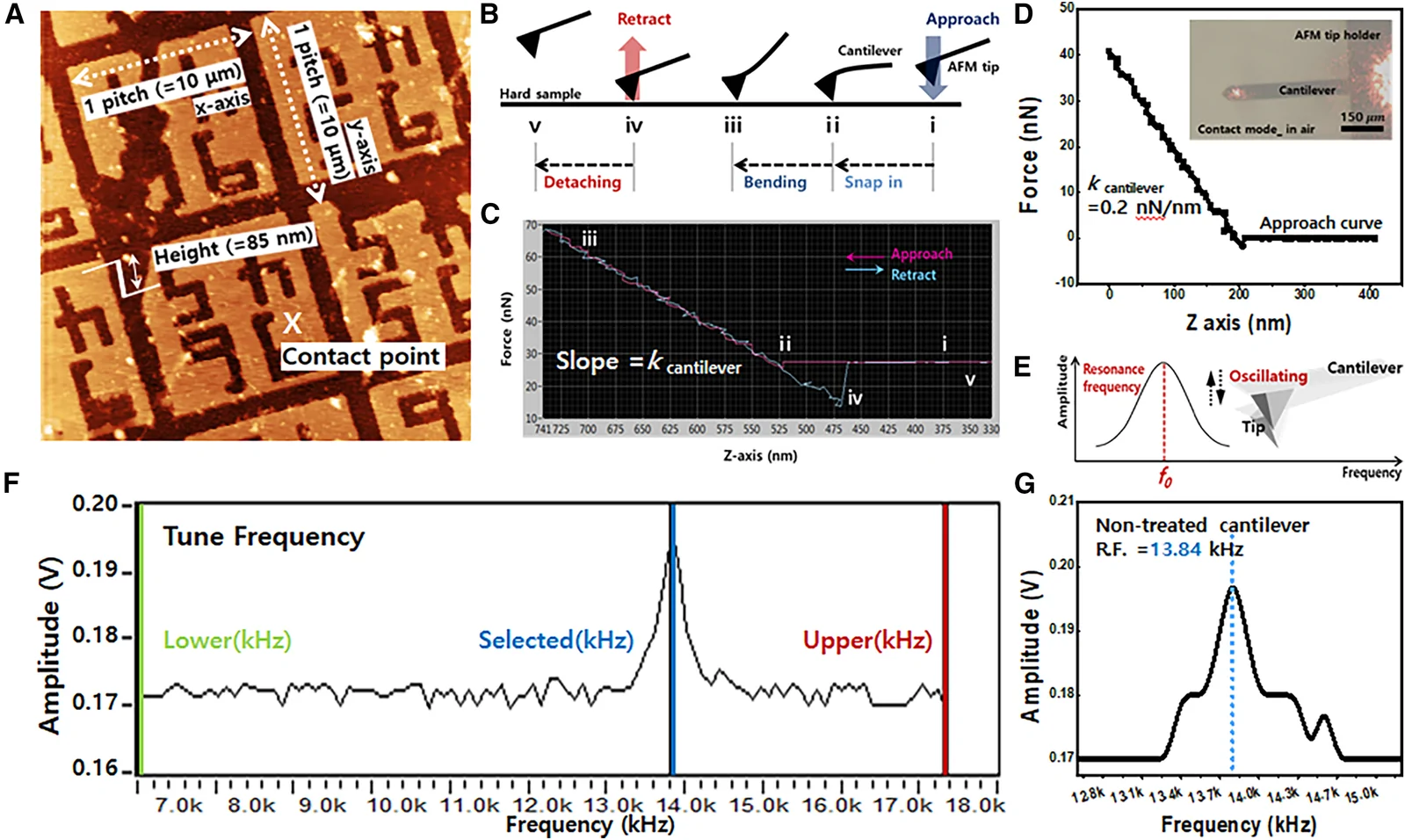
Optimization of resonance frequency measurement in air environments (A) Calibration of the fundamental length scales (*x*, *y*, and *z* axes) of the AFM topography system. (B and C) Force-distance curves illustrating the force exerted on the cantilever as a function of tip-sample separation, and (D) determination of the cantilever spring constant (inset: laser aligned on the backside of a contact-mode cantilever). (E) Measurement of resonance frequency through cantilever oscillation. (F) Frequency sweep process as displayed in the operating software of the AFM system, and (G) determination of the resonance frequency of a bare cantilever.

Although the spring constant of the cantilever is provided by the supplier, for precise experiments, the spring constant of the cantilever used in the experiment was directly measured and verified. The spring constant can be derived from changes in the force distribution related to the tip-sample distance over a hard surface, which corresponds to the FD curve (Figure 1B). When the tip contacts a hard sample surface, the applied load induces bending of the cantilever, allowing the spring constant to be calculated from the slope of the FD-curve (Figure 1C). The trace curve within the linear contact regime was used for spring constant extraction because it provided improved stability and reduced adhesion-related hysteresis effects. Under optimal conditions where the laser is well-aligned with the back of the cantilever, the FD-curve was measured. The measured spring constant value was 0.2 nN/nm, which perfectly matched the value provided by the AFM tip supplier (Figure 1D). The cantilever spring constant was determined from FD measurements performed under low-force loading conditions. A total of 50 times independent FD curves were acquired, and the spring constant was calculated from the linear contact region of the retrace curve. The average spring constant was determined to be k = 0.20119 ± 0.007379 nN/nm (*n* = 10). The maximum applied force was maintained below 5 nN to prevent cantilever damage during calibration.^31^^,^^32^^,^^33^ To verify probe integrity, resonance-frequency measurements and optical inspections were conducted before and after the calibration procedure, and no detectable changes in cantilever characteristics were observed.

The resonance frequency is a physical parameter obtained from cantilever vibrations in non-contact mode (Figure 1E). Therefore, the resonance frequency of the aluminum-coated cantilever was measured in non-contact vibration mode. By systematically adjusting the vibration mode, including amplitude, phase, and step resolution, we extracted the unique resonance peak of the cantilever (Figure 1F). As can be seen in the enlarged scan image, the resonant frequency of the cantilever was 13.84 kHz (Figure 1G) and this resonance profile is commonly observed in air-operated AFM systems due to instrumental transfer functions and environmental damping. This experimentally determined frequency showed close agreement with the nominal 13.00 kHz value provided by the cantilever supplier within experimental uncertainty.

### Calibration of AFM system parameters for resonance accuracy

During the acquisition of resonance spectra, the parameter number of steps (num steps), which defines the frequency resolution within a given sweep range was identified as a critical determinant of measurement accuracy. Calibration was therefore conducted using a non-contact oscillation probe to evaluate the influence of num steps on resonance detection (Figure 2A). At a constant sweep range, num steps were varied across four settings: 1,000, 200, 100, and 50. At high num steps (1,000, 200, and 100), the resonance curves exhibited a smooth, continuous profiles (Figures 2B–2D). Therefore, the maximum value of the resonant frequency can be clearly defined as shown in the enlarged resonance curve at num steps = 100 (Figure 2E). However, as the num steps decreased to 50, the resolution became insufficient to uniquely resolve the frequency peaks (Figure 2F). Since the frequency profile reaches a plateau rather than a distinct peak and then levels off, it is difficult to determine the frequency value at which the laser intensity is maximized as shown in enlarged image at num steps = 50 (Figure 2G). Intermediate num steps of 100 provided a balanced trade-off between resolution and acquisition time, yielding reliable and reproducible spectra. Based on these results, num steps of 100 was adopted as the optimized parameter for all subsequent resonance frequency measurements.

**Figure 2:**
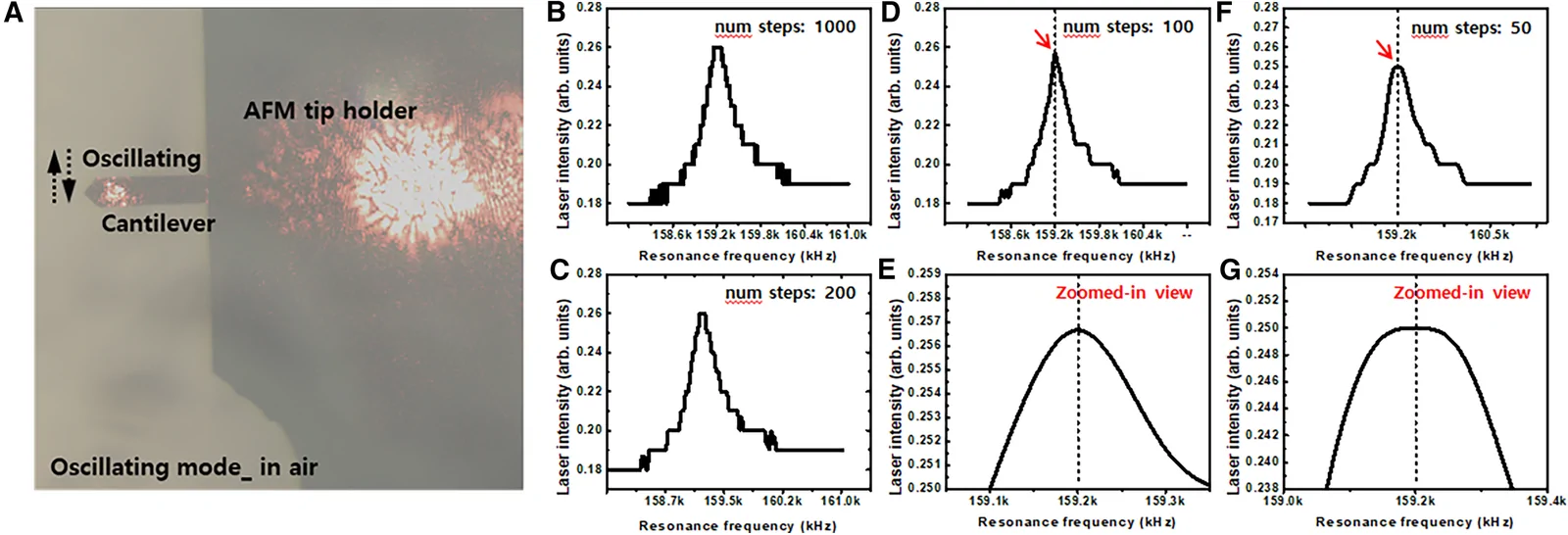
AFM system calibration parameters for resonance frequency (A) Measurement of cantilever resonance frequency in oscillation mode. Interface of the frequency sweep process showing adjustable frequency range and num steps (num steps) parameter. Resonance spectra obtained by sequentially decreasing num steps from (B) 1,000, (C) 200, (D) 100, to (F) 50. (E and G) Enlarged views of the resonance peaks for num steps = 100 and 50, respectively, for comparative analysis.

### Change in cantilever resonance frequency according to mass load

The frequency shift induced by mass loading on the cantilever was investigated. First, the influence of laser position on the resonance behavior of the cantilever was evaluated. As shown in Figure 3A, the resonance frequency was measured with the laser positioned at the cantilever tip (Figure 3B). The laser was subsequently repositioned to the midpoint of the cantilever, and the resonance frequency was measured again (Figures 3C and 3D). The results demonstrated that the laser position on the cantilever did not significantly affect the resonance frequency or vibration characteristics of the cantilever (Figures 3B and 3D). In contrast, when a resin droplet was deposited at the free end of the cantilever, a noticeable decrease in the resonance frequency was observed. During these measurements, the laser remained positioned at the midpoint of the cantilever (Figures 3B and 3D). These results indicate that the dynamic behavior of the cantilever is strongly influenced by the attached mass and that such mass loading can be quantitatively detected through resonance-frequency shifts.

**Figure 3:**
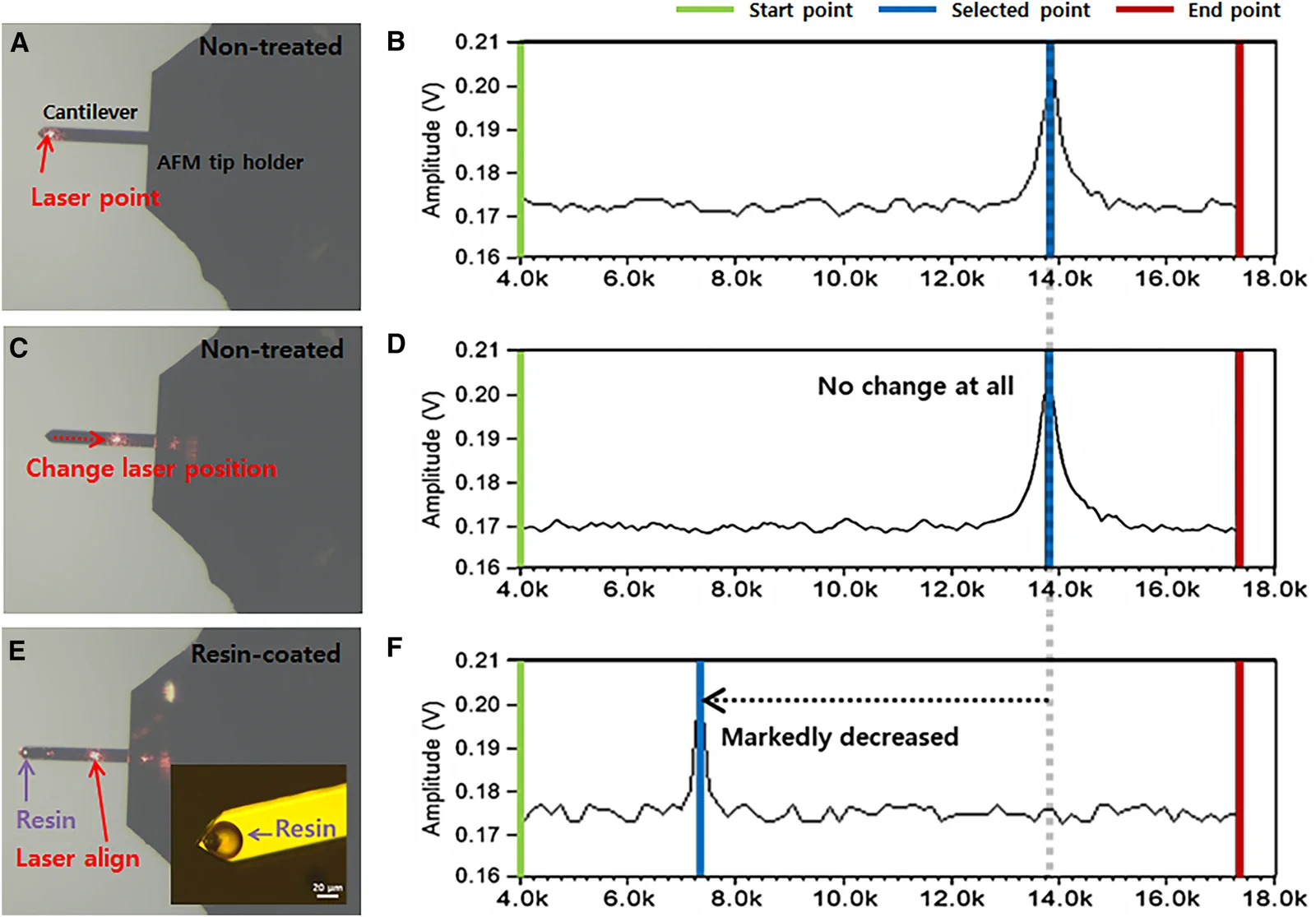
Mass-induced modulation of cantilever resonance frequency using resin coating (A and B) Laser alignment at the free end of the backside of a bare cantilever and measurement of its resonance frequency. (C and D) Comparison of resonance frequency values after moving the laser alignment from the cantilever tip toward the AFM tip holder region. (E) Laser alignment on a resin-coated cantilever (inset: resin deposited near the AFM tip region). (F) Downshift of the resonance frequency observed upon resin coating.

### Changes in resonant frequency according to resin particle mass

To systematically investigate the variation in resonant frequency with respect to the mass applied to a cantilever, resin droplets of varying sizes were attached to the cantilever. Droplets of different sizes were prepared by varying the amount of resin, placed on the cantilever, and cured by irradiating them with UV light of 9 W intensity for 60 s. The size of the resin droplets progressively increased, with the smallest having a cross-sectional area of approximately 108.13 μm^2^, increasing to 943.87, 1,094.67, and 2,703.17 μm^2^. Upon achieving complete curing of the resin, the resonant frequency of the cantilever was determined under the previously established conditions (Figure 4A).

**Figure 4:**
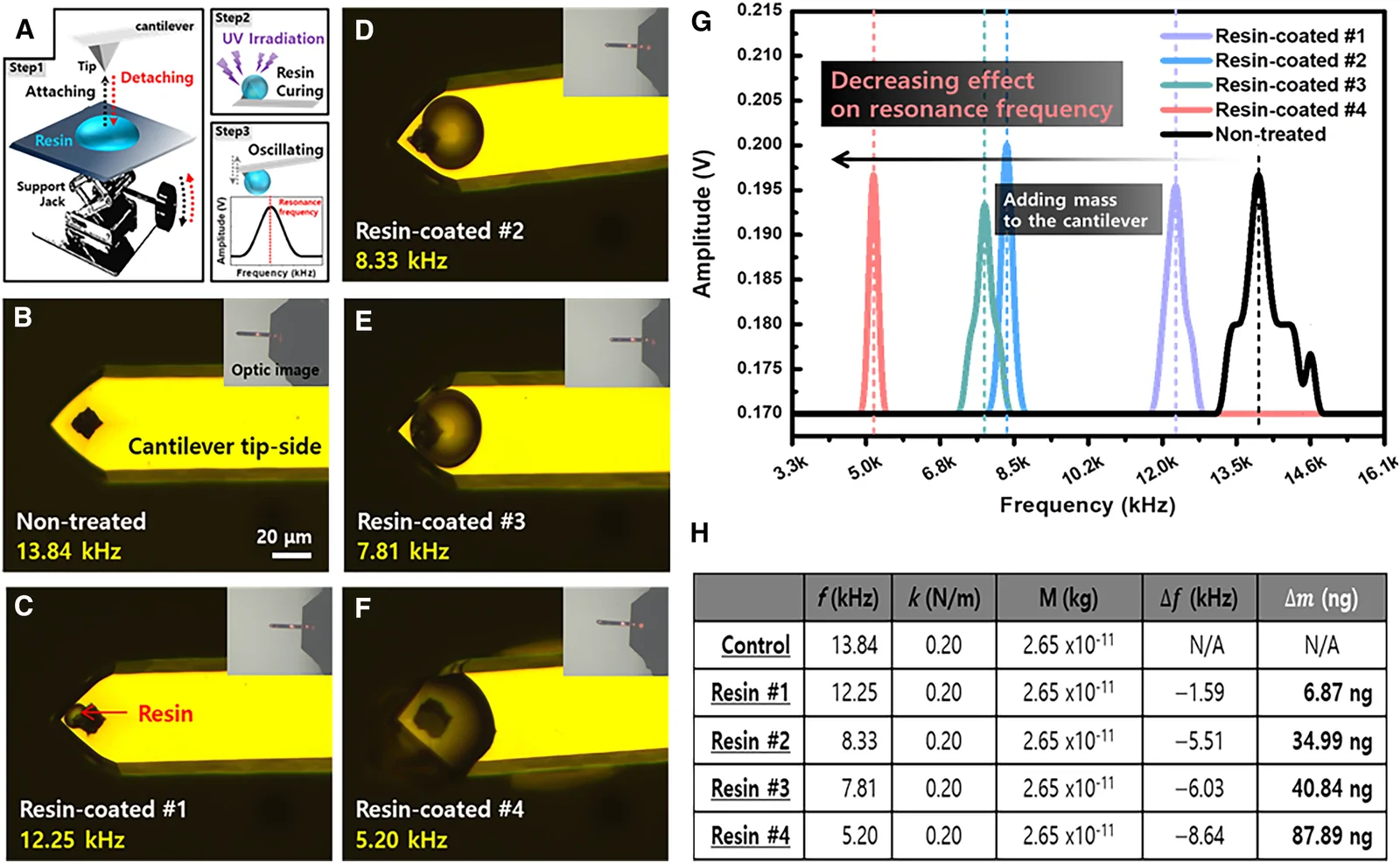
Relationship between resin particle size and cantilever resonance frequency shift (A) Resin coating process at the AFM cantilever tip region. Resonance frequencies of five cantilevers were compared: optical images of (B) non-treated and (C–F) resin-coated samples #1–#4 (inset: optical image of a laser-aligned cantilever). (G) Comparison of resonance frequency downshifts between non-treated and resin-coated cantilevers. (H) Calculation equivalent mass of resin attached to cantilevers based on experimental resonance effective frequencies of resin-coated samples.

Although precise control of the position of the resin droplets is challenging, every effort was made to position them at the tip of the cantilever. The attachment location and size of the resin droplets were confirmed using an optical microscope. As shown in Figures 4C–4F, the difference in size of the resin droplets is very distinct. The resin attached samples were labeled sequentially from smallest to largest as sample #1, #2, #3, and #4.

As shown in Figure 4G, the vibration frequency of the cantilever decreased as the size of the resin droplets increased. The control group’s frequency was 13.84 kHz, and it shifted to 12.25 kHz with the smallest resin droplet (#1). As the size of the resin droplet increased to 943.87 μm^2^ (#2), the frequency decreased rapidly to 8.33 kHz, and samples #3 and #4 vibrated at low frequencies of 7.81 kHz (#3) and 5.20 kHz (#4), respectively. Since an increase in the size of the resin droplet directly indicates an increase in its mass, this result suggests that changes in the cantilever vibration mode due to mass changes can be detected. In particular, as seen in samples #2 and #3, it suggests that changes in the cantilever vibration mode due to minute mass differences can also be detected.

The interaction between the cantilever and the resin droplet can be described using *mass-spring theory*. A mass-spring system is a mechanical model that explains the behavior of a mass attached to a spring.^27^ The spring follows Hooke’s law, exerting a force proportional to the displacement from its equilibrium position. This system exhibits simple harmonic motion, where the mass oscillates back and forth around the equilibrium position once released from displacement.

According to *mass-spring theory*, the mass of the resin droplet can be calculated using the resonant frequency detected in Figure 4G and the spring constant of the cantilever set to a calibrated value of 0.2 N/m. First, the effective mass of the cantilever was calculated using the resonant frequency obtained in the bare state (13.84 kHz), and a value of approximately 26.5 ng was obtained. Next, the masses of samples #1, #2, #3, and #4 were calculated, and the masses of the resin droplets were determined to be 6.87, 34.99, 40.84, and 87.89 ng, respectively (Figure 4H).

### COMSOL simulation for verification of nanoscale mass quantification

To verify the accuracy of the nanogram-scale masses estimated from the mass-spring theory, finite element simulations were conducted using COMSOL Multiphysics. The simulations were based on the experimentally measured resonance-frequency shifts resulting from resin-droplet loading on the cantilever.

The simulations were performed under the following conditions. (1) The system was configured to enable three-dimensional testing of eigenfrequency analysis within the framework of solid mechanics. (2) The geometric parameters of the contact mode cantilever were set to 450 × 50 × 2 μm^3^ for length, width, and depth, respectively (Figure 5A). This is the same dimension as the cantilever used in the experiment. The dimensions of the AFM cantilevers were verified using optical microscopy prior to experimentation. Measurements obtained from eleven independently selected cantilevers yielded average dimensions of 449.6 ± 1.0 μm in length, 49.7 ± 0.5 μm in width, and 2.04 ± 0.04 μm in thickness (mean ± SEM, *n* = 11). These values were consistent with the manufacturer’s nominal specifications of 450 × 50 × 2 μm^3^. (3) The material properties of the cantilever were modeled as a silicon-based solid structure with the following material properties: density of 2,329 kg/m^3^, elastic modulus of 17 × 10^7^ kPa, and Poisson’s ratio of 0.28.^34^^,^^35^^,^^36^ (4) Boundary conditions were defined such that one end of the cantilever was fixed and the other end was designated as a free boundary supporting the load (Figure 5B). (5) The cantilever was affected by atmospheric pressure and gravity, which were −1,013.25 hPa and −9.8 m/s^2^, respectively (Figure 5B). (6) A uniform mesh was firstly generated using a parametric modeling tool (sequence type: physics control mesh), which automatically generates a mesh based on user-defined parameters. To improve accuracy, the mesh density at the free end was increased to element size of extremely fine in COMSOL software (Figure 5C). In cantilever bending process, the magnitude of the stress distributed along its length was visualized using a color gradient. The fixed region (red) experienced relatively higher stress compared to the point of applied force (blue) (Figure 5D). The point-mass approximation was intentionally adopted as a first-order simplification to isolate the dominant inertial loading effect on resonance frequency. The simulated fundamental resonance frequency was 13.00 kHz, and this value can be regarded as highly reliable, as it is in close agreement with the theoretical eigenfrequency of the employed contact-mode cantilever (Figure 5E). This close agreement supports the validity of the computational model and the overall experimental methodology. The relationship between additive resin mass and the corresponding frequency shift was subsequently visualized graphically by using OriginPro (v.8.5, OriginLab Corp., USA). And also, we performed data fitting based on a logistic model for quantitative analysis. (Figure 5F).

(Equation 1) $$y=A_{2}+\frac{\left(A_{1}-A_{2}\right)}{\left\{1+{\left(\frac{x}{x_{0}}\right)}^{p}\right\}}.$$

**Figure 5:**
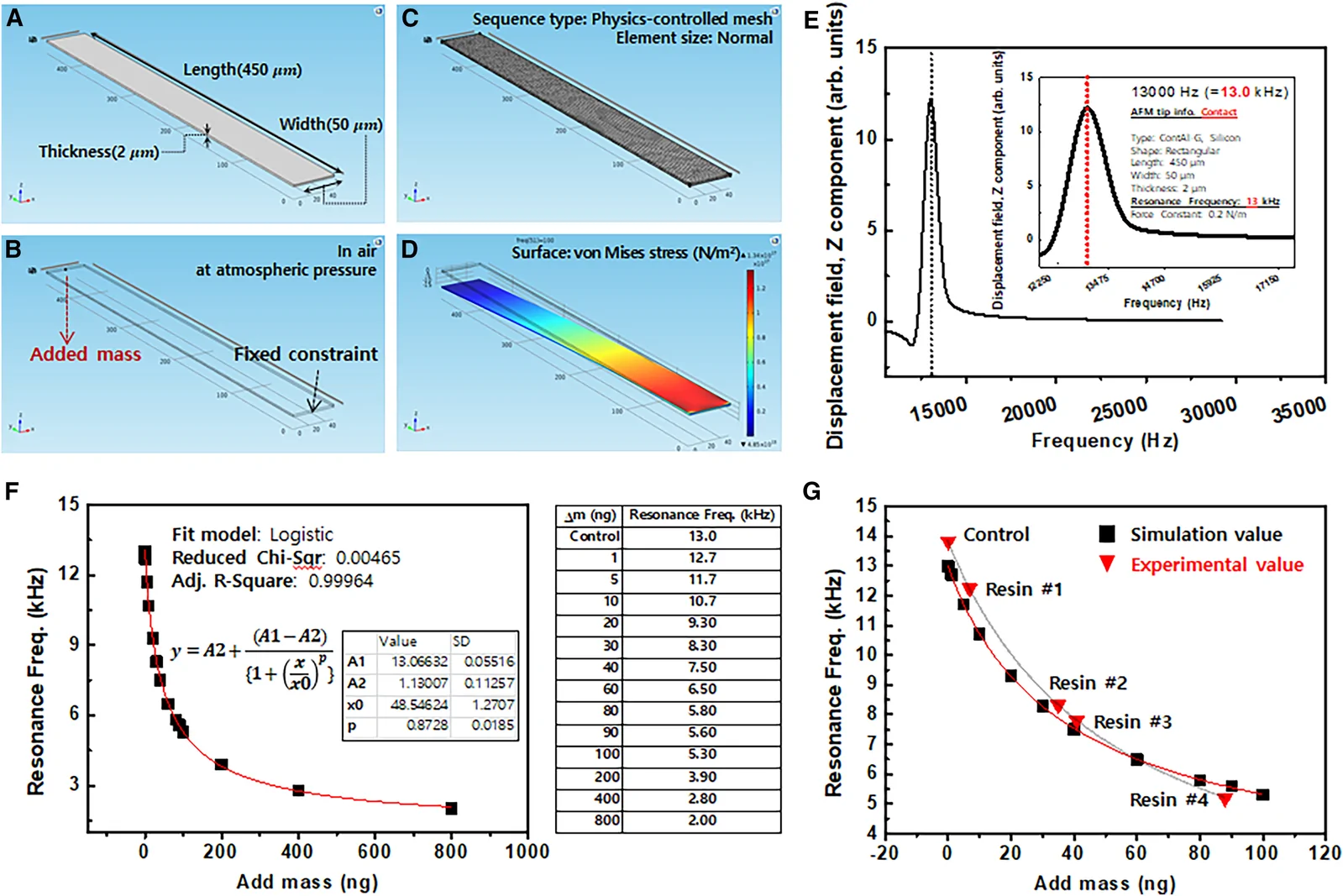
Computational simulation of resin mass-induced frequency shifts (A–D) Virtual AFM cantilever modeled in COMSOL Multiphysics with experimental setup parameters, including cantilever width, depth, height, atmospheric pressure conditions, added mass location, fixed boundary, and mesh element size. (E) Simulated resonance frequency of the virtual cantilever. (F) Simulated resonance frequency shifts corresponding to added mass at the cantilever tip. (G) Comparison between experimental and simulated resonance frequency values.

The fitted curve showed excellent agreement with the simulation data, as indicated by a reduced chi-square of 4.65 × 10^−3^ and an adjusted R-square of 1.00. The fitted parameters were A_1_ = 13.07 ± 0.06, A_2_ = 1.13 ± 0.11, *x*_0_ = 48.55 ± 1.27, and *p* = 0.87 ± 0.02. And the fitting results with the experimentally measured data, reduced chi-square of 1.03 × 10^−13^ and an adjusted R-square of 1.00. The fitted parameters were A_1_ = 13.84 ± 3.21 × 10^−7^, A_2_ = 1.14 ± 1.58 × 10^−5^, *x*_0_ = 52.90 ± 1.27 × 10^−4^, and *p* = 1.00 ± 1.21 × 10^−6^. Both the simulation-derived (black squares) and experimentally measured (red triangles) datasets were fitted to a logistic model, and a consistent trend was observed in which the resonance frequency of the cantilever decreased as the vertically added mass increased (Figure 5G).

## Discussion

This study demonstrates that AFM can be utilized beyond its role as a high-resolution imaging tool to perform precise nanoscale mass analysis. It was found that rigorous calibration of the AFM cantilever is crucial for accurate measurement of minute masses. Initially, the dimensions and mechanical properties of the cantilever were thoroughly verified. This process helped minimize potential errors from geometric distortion or cantilever flexibility during measurements. Specifically, the spring constant of the cantilever was measured through FD-curve measurements, and these values were consistent with the specifications provided by the supplier, confirming the accuracy of the experimental conditions and the stability of the equipment. Similar calibration considerations have been reported in previous AFM-based quantitative characterization studies, where spring-constant accuracy was identified as a major factor governing measurement reliability.^37^^,^^38^

Additionally, non-contact oscillation measurements further confirmed the validity of this approach. The experimentally obtained resonance frequency closely matched the theoretical predictions and fell within the expected error range for cantilever-based systems. The discrepancies can be attributed to structural differences in the cantilever, material coating, and environmental damping effects, which can induce subtle changes in resonance behavior. Meanwhile, the sharp resonance peaks observed in the enlarged resonance frequency spectrum image indicate that external disturbances, such as viscous damping and alignment errors, were effectively eliminated through optimization of the cantilever conditions. Therefore, accurately measuring the resonance frequency of an AFM cantilever requires both system calibration and spring constant verification. This enables precise mass measurement at the nanoscale using AFM.

Beyond the resonance frequency setting, further research confirmed that acquisition parameters, particularly the number of frequency steps, significantly impact the quality and interpretability of resonance spectra. Lower step values resulted in increasingly smoother spectral profiles, demonstrating an inherent trade-off between acquisition efficiency and spectral resolution. Below a certain threshold, resonance peaks became indistinguishable, compromising the reliability of resonance-based quantification. Optimizing parameters with 100 steps improved measurement stability and provided a reproducible standard that ensured compatibility between AFM setups. These results highlight the need for rigorous calibration of data acquisition parameters. While often overlooked in routine AFM practice, this is crucial for ensuring the reproducibility and comparability of nanoscale measurements.

Subsequent experiments focused on measuring changes in AFM cantilever resonance due to nanoscale mass. Initially, mass was added by coating the cantilever with polydimethylsiloxane (PDMS). As expected, the resonant frequency of the PDMS-coated cantilever was significantly lowered. However, due to the high viscosity and adhesive properties of PDMS, selective and localized coating was technically unfeasible. This resulted in unwanted coverage across the entire cantilever surface, introducing unexpected variability (Figure S1).

So, resin droplets were used as mass-loading substitutes. The present study specifically employed resin droplets because they enabled controllable localized mass loading using a simple and accessible experimental workflow compatible with conventional AFM systems. These droplets allowed us to control their size and attachment location, and systematically analyze the mass-frequency relationship under controllable conditions. Many studies have demonstrated cantilever-based mass calibration using reference objects with well-defined geometries and material properties, such as microspheres, nanoparticles, and suspended microchannel resonators, enabling the added mass to be independently determined prior to or during measurement.^39^^,^^40^^,^^41^ In contrast, the present study intentionally employed UV-curable resin droplets because they provide a simple, inexpensive, and experimentally accessible means of generating localized mass loading on a conventional AFM cantilever without requiring specialized micromanipulation or microfluidic systems. However, unlike calibrated particles, the deposited resin droplets do not possess predefined volumes or geometries. Although their projected cross-sectional area can be measured optically, their three-dimensional morphology, contact angle, and polymerization-induced shape variations prevent accurate determination of droplet volume and mass based solely on geometric measurements. Therefore, the added mass was not estimated from the droplet dimensions or material density but was indirectly determined by correlating experimentally measured resonance frequency shifts with finite element simulations performed using identical cantilever geometry and boundary conditions. The close agreement between the experimental results and COMSOL simulations supports the validity of this indirect mass determination approach over the investigated nanogram-scale mass range.

The resonance frequency of the resin-coated cantilever decreased significantly compared to the control, confirming that even very small mass changes can be sensitively detected by AFM resonance spectroscopy. Particularly interesting results emerged from an analysis of the cantilever-coupled resin particle size. The tendency for larger resin particles to attract greater resonant predictions confirms the validity of the harmonic oscillator model at the nanoscale. Importantly, these results go beyond mere proof-of-concept and demonstrate that the resonant frequency shift is qualitatively and quantitatively correlated with the size of the added mass. The present analysis assumes an idealized point mass located at the free end of the cantilever, whereas actual resin droplets possess finite dimensions and a spatially distributed mass profile. As a result, distributed loading may modify the effective modal participation and slightly alter the resonance-frequency response compared with the idealized lumped-mass assumption. In addition, small deviations of the droplet position from the cantilever tip may reduce mass sensitivity because the modal displacement amplitude decreases away from the free end.

The practical detection limit of resonance-based cantilever mass sensing is governed by the minimum resolvable frequency shift, effective modal mass, and environmental noise sources. Based on the experimentally achieved frequency resolution and the effective cantilever mass model, the detectable mass variation in the present study was estimated to lie within the nanogram (6.87–87.89 ng; see Figure 4H) range under optimized ambient conditions. Based on the finite-element simulation results, measurable resonance-frequency shifts were observed over an added mass range of approximately 1–800 ng, indicating the potential capability of the cantilever to detect nanogram-scale mass variations within this range. This sensitivity is consistent with previously reported microcantilever-based mass sensors operating in air,^42^ which typically achieve detection limits ranging from hundreds of picograms to several nanograms depending on resonance stability and measurement noise.^43^

Through experiments with resin particles, we demonstrate that AFM-based resonance analysis can be used to measure the mass of cell aggregates or spheroids, providing a foundation for extending this approach to more complex biological systems. Perhaps the most remarkable aspect of this study lies in the integration of experimental measurements and computational modeling of resonance frequencies. Simulations that replicate the geometric and material properties of the AFM cantilever yielded resonance frequencies that matched the frequency derived from measurement and theory. This agreement clearly demonstrates the accuracy of the simulation parameters. More importantly, this simulation enables precise calculations of nanogram-scale masses using a mass-spring system. The ability to correlate resonance frequency shifts with effective resonance-equivalent masses supports the utility of AFM as a nanoscale resonance-based mass analysis tool. Furthermore, the established mass-frequency relationship allows researchers to accurately predict unknown mass values from experimental results.

Because the experimentally investigated mass range produced only minor structural deformation of the cantilever, nonlinear geometric effects were expected to be negligible. Consequently, the use of linear eigenfrequency analysis provided an appropriate approximation for evaluating resonance-frequency shifts associated with mass loading.

This study highlights that AFM-based resonant frequency analysis can serve as a powerful approach for nanoscale mass detection. By systematically addressing methodological issues such as calibration, environmental stability, coating strategies, and computational modeling, we have established a clear experimental and theoretical framework that ensures reliability and reproducibility. The ability to detect and quantify minute mass changes at the nanogram level opens intriguing application possibilities, particularly in biological research evaluating the physical properties of three-dimensional cell spheroids, tumor microtissues, or other microassemblies. This approach may also be useful in materials science, where accurately characterizing the mass loading and mechanical resonance of nanostructures is increasingly important. Future research will focus on improving selective coating methodology for more precise control of mass deposition and expanding the computational framework to include viscoelasticity and heterogeneous material properties. Ultimately, integrating AFM resonance spectroscopy with advanced modeling techniques will establish a new paradigm for nanoscale mass measurement, bridging fundamental biophysics with innovative biomedical applications.

The present results establish a foundation for resonance-based nanoscale mass sensing using AFM cantilevers. While the methodology may ultimately prove useful for biological applications, further investigation under biologically relevant experimental conditions is required before direct application to cells, tissues, and tumor spheroids can be confirmed.

### Limitations of the study

Several limitations of the present study should be considered when interpreting the results. First, resin droplets were used as model mass-loading materials rather than biological samples or calibrated reference particles. Although the resin droplets provided a practical and controllable means of generating localized mass loading, their irregular three-dimensional morphology and variable contact geometry prevented direct determination of their mass from geometric measurements. Consequently, the added mass was indirectly estimated by correlating experimentally measured resonance-frequency shifts with finite element simulations. Although the close agreement between experimental measurements and simulations supports the validity of this approach, the use of an indirect mass determination method may introduce uncertainties that should be further evaluated using reference materials with independently characterized masses. Second, the current analysis was based on an idealized lumped-mass model in which the added mass was assumed to be concentrated near the cantilever tip. In practice, the mass of a deposited object is spatially distributed, and its position and geometry may influence modal participation and resonance-frequency shifts. Future studies incorporating distributed mass loading and experimentally determined deposition locations may therefore improve quantitative accuracy. Third, the estimated detection range was obtained under optimized ambient conditions and may not directly represent the detection performance under biologically relevant environments. Factors such as fluidic damping, temperature fluctuations, surface interactions, and sample attachment may substantially affect resonance stability and mass sensitivity. Finally, although the results demonstrate nanogram-scale mass detection using AFM resonance analysis, direct validation with cells, spheroids, or other biological specimens was beyond the scope of this study. Further studies using biologically relevant samples and independently calibrated mass standards will be necessary to establish the accuracy, reproducibility, and applicability of this approach for quantitative biological mass measurements.

## Resource availability

### Lead contact

Further information and requests for resources and reagents should be directed to and will be fulfilled by the lead contact, Sangwoo Kwon (tereus7@naver.com).

### Materials availability

This study did not generate new unique reagents or biological resources. All materials used in this study were commercially available or otherwise described in STAR Methods.

### Data and code availability

• The datasets generated and analyzed during this study, including the AFM resonance frequency measurements and mass-loading data are available from the lead contact upon reasonable request.
• This study did not generate original code. The software used for data analysis and finite-element simulations is described in STAR Methods.
• Additional information required to reproduce or reanalyze the results reported in this paper is available from the lead contact upon reasonable request.

## Acknowledgments

This research was supported by the 10.13039/501100003725National Research Foundation of Korea (NRF) (grant number RS-2024-00352605), funded by the Korean Government (10.13039/501100014188MSIT).

## Author contributions

S.K., writing – original draft, methodology, data curation, and conceptualization. K.S.K., writing – original draft, review and editing, and supervision.

## Declaration of interests

The authors declare that they have no known competing financial interests or personal relationships that could have appeared to influence the work reported in this paper.

## STAR★Methods

### Key resources table

**Table:** 

REAGENT or RESOURCE	SOURCE	IDENTIFIER
**Software and algorithms**		

COMSOL Multiphysics 5.0	COMSOL Inc.	Version 5.0
OriginPro 8.5	OriginLab Corp.	Version 8.5

**Other**		

AFM system	AFMWorkshop Inc.	TT-AFM, CS3230
Optical microscope	ZEISS	NA806-LQD
ContAl-G cantilever	BudgetSensors®	ContAl-G
PR-T190 cantilever	BudgetSensors®	PR-T190
UV-curable resin	PastelCraft Inc.	아-01–111

### Method details

#### Cantilever preparation

Before experiments, the AFM cantilevers (ContAl-G and PR-T190, BudgetSensors®, Bulgaria) underwent a rigorous cleaning protocol to remove any organic residues or particulate contaminants that could compromise measurement accuracy. The cleaning procedure consisted of sequential immersion in analytical-grade acetone(5 min), isopropanol(5 min), and deionized water(5 min, 3 times), each step performed under sterile conditions to minimize contamination. After solvent treatment, the cantilevers were thoroughly dried under a high-purity nitrogen gas flow and stored in a dust-free, environmentally controlled chamber until further use. Baseline characterization was performed by measuring the natural resonant frequency of each cantilever in an ambient environment using both contact and non-contact (tapping) modes of the AFM system (Table Top, AFMWorkshop Inc., USA). Specifically, the spring constant of the cantilevers was quantitatively determined through FD curve analysis. This step was used to establish a baseline frequency spectrum prior to resin deposition.

#### Resonance frequency measurement

The resonant frequency of the cantilever was measured under air conditions using non-contact AFM mode. The cantilever was excited using the built-in piezoelectric actuator, and resonance spectra were acquired over a frequency range encompassing the first flexural resonance mode. Frequency sweeps were conducted using a step size of 0.01 kHz while maintaining a constant excitation amplitude. The resonance frequency was determined from the maximum amplitude position of the resonance spectrum. Each measurement was repeated 50 times, and the average value was used for subsequent analysis. All measurements were performed under identical environmental conditions to minimize variations arising from temperature fluctuations and instrumental drift.

#### Cantilever resonance frequency modulation through resin mass loading

To systematically investigate the correlation between cantilever resonance frequency and applied mass, different volumes of resin droplets were deposited at the ends of tip of each cantilever. Resin deposition was performed under an optical microscope to control droplet placement and uniformity. The resin volume was precisely adjusted for each cantilever to establish a gradient in the mass-loading condition. After deposition, the resin was photopolymerized by exposure to 9 W ultraviolet (UV) light for 1 min. Curing parameters were optimized to minimize shrinkage artifacts and density inhomogeneities. The deposited resin droplets exhibited horizontal diameters ranging approximately from 10 to 70 μm, as determined by optical microscopy. Representative droplet geometries were characterized prior to resonance measurements to ensure consistent mass-loading conditions among samples. The deposited resin droplets were not fabricated as standardized calibration masses but rather as experimentally generated localized mass loads. Subsequently, frequency sweep analysis was performed to record changes in resonant frequency. Both amplitude and phase response curves were collected to characterize the cantilever dynamics under various loading conditions. This systematic approach allowed us to quantitatively establish the mass-frequency relationship.

#### Numerical simulation of mass-induced frequency shifts using COMSOL multiphysics

Computational modeling was performed using Finite element analysis (FEA) to simulate the effect of mass changes on the cantilever resonant frequency. FEA was conducted using COMSOL multiphysics (version 5.0.0, COMSOL Inc., USA). The model was established using the Model Wizard by selecting a three-dimensional (3D) geometry and the Solid Mechanics interface within the Structural Mechanics module. Eigenfrequency analysis was subsequently performed to evaluate the resonance characteristics of the cantilever. A physics-controlled mesh was applied throughout the computational domain, and the mesh was uniformly set to “Fine,” corresponding to a maximum element size of approximately 36 μm and a minimum element size of approximately 4.5 μm. The mesh element size was uniformly specified as Fine (element quality: 0.86, number of elements: ∼45,000) for all simulations to maintain numerical consistency and accuracy.

The cantilever was modeled as an elastic beam with boundary conditions corresponding to a fixed-free configuration. Mass loading was implemented by introducing point masses at the free end of the cantilever, mimicking resin deposition or nanoparticle adhesion. A series of discrete point mass values (*m*_1_, *m*_2_, *m*_3_ …) were assigned to evaluate the resonant frequency shift. A fine finite element mesh was used to ensure numerical accuracy, and additional refinement was performed to capture local perturbations near the loaded region. Natural frequency analysis was performed to extract the fundamental and higher-order resonant modes. The simulation data were compared with analytical predictions derived from a simplified mass-spring model.^44^

(Equation 2) $$f=\frac{1}{2\pi }\sqrt{\frac{k}{m_{eff}}}$$

where *k* is the effective spring constant of the cantilever and *m*_*eff*_ represents the effective modal mass of the system. Modal mass is the mass associated with a specific mode of vibration in a system and represents how much of the total mass participates in that mode. Here, this refers only to the mass of the cantilever.

#### Quantification of resin mass through resonant frequency shifts

Experimental quantification of the resin mass was performed by comparing the resonant frequency spectra of the cantilevers before and after resin deposition. Measurements were performed using an AFM non-contact vibration mode to track the frequency shift induced by the bonded resin. The resonant frequency of each unloaded cantilever was used as a baseline, and the frequency of the cantilever subjected to resin loading was compared against this baseline. The frequency downshift was translated into an equivalent mass change using the following relationship^44^:

(Equation 3) $$\Delta m=-2\frac{m_{eff}}{f}\Delta f$$

where Δ*f* denotes the frequency shift, *m*_*eff*_ is the effective cantilever mass, *f* is the baseline resonance frequency, and Δ*m* corresponds to the calculated resin mass. By systematically applying this approach across resin droplets of varying sizes, a quantitative relationship between resin mass and frequency response was established.

### Quantification and statistical analysis

Resonance frequency measurements were repeated 50 times under identical experimental conditions, and the mean value was used for subsequent analysis. Here, *n* = 50 denotes the number of repeated resonance-frequency measurements and does not represent the number of independent experimental samples. Data are presented as mean ± standard error of the mean (SEM), where applicable. The relationship between added mass and resonance frequency was analyzed by logistic model fitting using OriginPro 8.5 (OriginLab Corp., USA). Goodness of fit was evaluated using the reduced chi-square and adjusted R-square values. Finite-element eigenfrequency simulations were performed using COMSOL Multiphysics 5.0 (COMSOL Inc., USA), and the simulated resonance frequencies were compared with the experimentally measured values. No inferential statistical tests were performed because the study was designed to characterize resonance-frequency responses, quantify the relationship between mass loading and resonance frequency, and compare experimental measurements with computational simulations, rather than to test differences between independent experimental groups. Statistical and quantitative analysis details are provided in the corresponding figure legends and in the results and STAR Methods sections, where applicable.
