# Supplementary material for: A systematic approach to quantitative nanoscale mass spectrometry using atomic force microscopy
Source: iScience. 2026 Oct 15;29(11):117900. doi: 10.1016/j.isci.2026.117900 (PMC13694898; doi:10.1016/j.isci.2026.117900)
Supplement: Supplementary file 1 [file mmc1.pdf]

iScience, Volume *29*

Supplemental information

A systematic approach to quantitative nanoscale mass spectrometry using atomic force microscopy

Sangwoo Kwon and Kyung Sook Kim

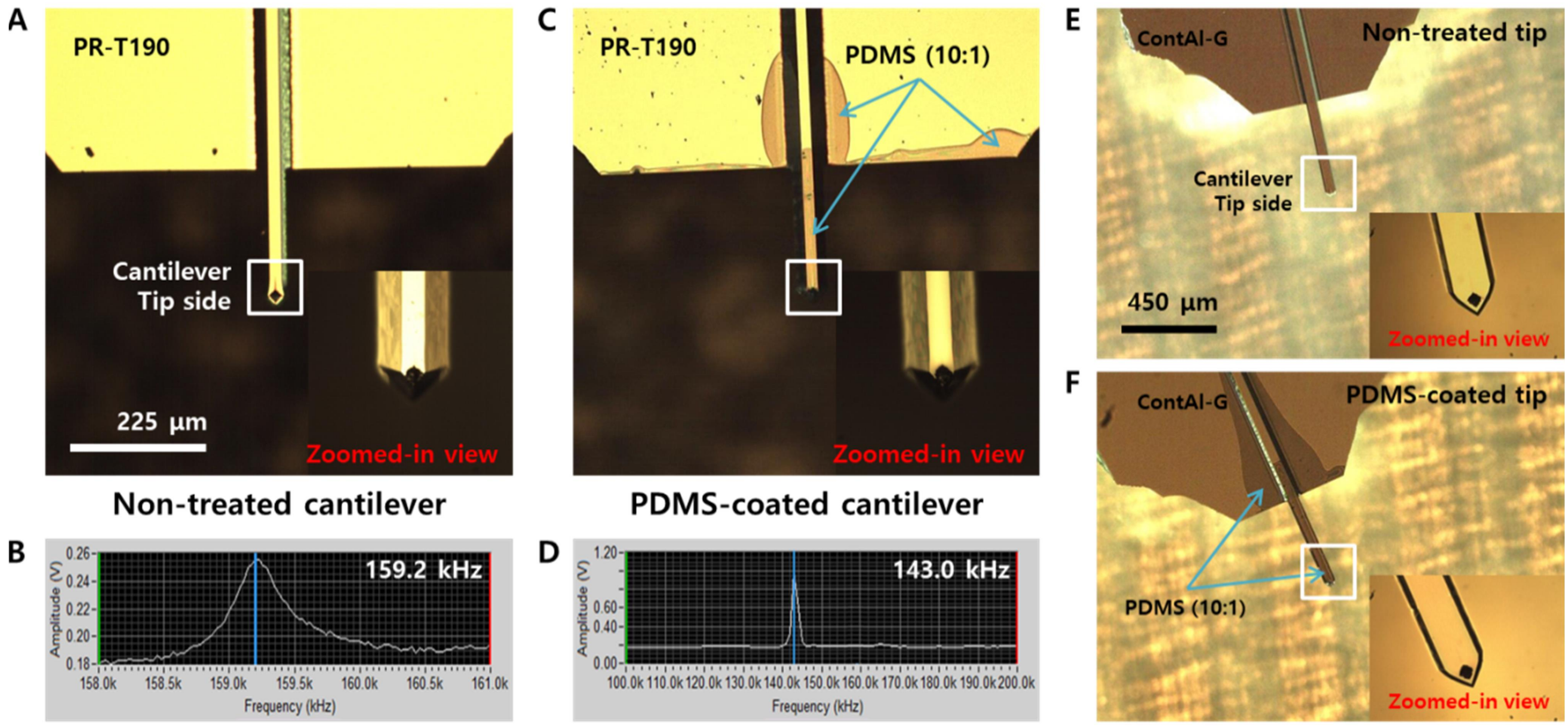

Fig S1. Preliminary Evaluation Using Polydimethylsiloxane (PDMS) Coating. As an initial strategy for modulating resonance frequency, PDMS was coated onto AFM cantilevers (10:1 mixing ratio of base to curing agent) (Fig. S1A, S1C). Application of PDMS induced a substantial resonance frequency reduction of approximately 15 kHz (Fig. S1B, S1D). However, due to its inherent viscosity and adhesive properties, PDMS was observed to spread uncontrollably, covering not only the tip but also the shaft and backside of the cantilever. Multiple attempts were made to achieve selective deposition by modifying coating volume and curing parameters, yet consistent overcoating persisted. To overcome this, longer cantilevers designed for contact mode operation nearly double the length of standard non-contact probes were tested under the hypothesis that the extended geometry would facilitate localized deposition near the tip. Despite these modifications, selective confinement of PDMS to the distal tip remained unattainable (Fig. S1E–F). This outcome highlighted the limitations of PDMS for controlled, localized mass loading, thereby justifying the subsequent adoption of resin-based approaches.
